# Equalization Optimizer-Based LSTM Application in Reservoir Identification

**DOI:** 10.1155/2022/7372984

**Published:** 2022-09-09

**Authors:** Fan Yang, Kewen Xia, Shurui Fan, Zhiwei Zhang

**Affiliations:** Hebei University of Technology, College of Electronic Information Engineering, Tianjin 300401, China

## Abstract

In recent years, the use of long short-term memory (LSTM) has made significant contributions to various fields and the use of intelligent optimization algorithms combined with LSTM is also one of the best ways to improve model shortcomings and increase classification accuracy. Reservoir identification is a key and difficult point in the process of logging, so using LSTM to identify the reservoir is very important. To improve the logging reservoir identification accuracy of LSTM, an improved equalization optimizer algorithm (TAFEO) is proposed in this paper to optimize the number of neurons and various parameters of LSTM. The TAFEO algorithm mainly employs tent chaotic mapping to enhance the population diversity of the algorithm, convergence factor is introduced to better balance the local and global search, and then, a premature disturbance strategy is employed to overcome the shortcomings of local minima. The optimization performance of the TAFEO algorithm is tested with 16 benchmark test functions and Wilcoxon rank-sum test for optimization results. The improved algorithm is superior to many intelligent optimization algorithms in accuracy and convergence speed and has good robustness. The receiver operating characteristic (ROC) curve is used to evaluate the performance of the optimized LSTM model. Through the simulation and comparison of UCI datasets, the results show that the performance of the LSTM model based on TAFEO has been significantly improved, and the maximum area under the ROC curve value can get 99.43%. In practical logging applications, LSTM based on an equalization optimizer is effective in well-logging reservoir identification, the highest recognition accuracy can get 95.01%, and the accuracy of reservoir identification is better than other existing identification methods.

## 1. Introduction

With the development of logging technology, the interpretation technology associated with it is gradually moving from qualitative and quantitative manual processing to the era of quantitative processing using machines. Traditional reservoir identification mainly relies on expert experience, construction of rendezvous plates, and other methods, but the conventional reservoir identification methods are subject to many human influence factors. Currently, increasingly scholars are proposing the use of artificial neural networks to solve the reservoir identification problem, thus effectively avoiding errors and improving production efficiency [1–3]. This study provides a basis for reservoir quality and oil-bearing evaluation of terrestrial shale reservoirs. However, these methods ignore the time-series nature of logging data and do not conform to the practical geological thinking and the logic of traditional geological analysis.

The reservoir data are temporal in nature, with strong backward and forward correlation, so the use of long short-term memory networks is considered for the processing of reservoir data. Long short-term memory (LSTM) is a special type of RNN that can solve the gradient explosion and gradient disappearance problems during the training of long sequences. They can also improve the performance of long sequences. At present, some scholars use LSTM to identify reservoirs. For example, Zhou et al. [4] established a Bi-LSTM network model, which can accurately identify different types of strata developed in storage space and significantly improve the accuracy of reservoir identification. Chen et al. [5]constructed a multilayer LSTM for fine reservoir parameter prediction. The results show that multilayer LSTM has better robustness and accuracy in prediction.

In recent years, LSTM has made breakthroughs in many fields, and the use of intelligent optimization algorithms combined with LSTM is also one of the best ways to improve model shortcomings and increase classification accuracy. For example, Xie et al. [6] used the enhanced gray wolf optimization(GWO) algorithm for the CNN-LSTM model of time-series prediction and indicated that the classification accuracy was improved. Peng et al. [7] applied the fruit fly algorithm (FOA) to optimize the hyperparameters of the LSTM neural network, and the results showed that the prediction accuracy of the FOA-LSTM model was greatly improved. Yang et al. [8] build an improved lion swarm algorithm (LSO) for the LSTM to optimize the hyperparameters of the LSTM model, and the results showed that the enhanced model has strong generalization ability and higher prediction accuracy. When using the LSTM model for reservoir identification, various parameters of the LSTM need to be artificially selected, resulting in insufficient accuracy in reservoir identification. A strategy is proposed to optimize the number of neurons and hyperparameters of the LSTM model by gradient descent using an improved equilibrium optimizer.

With the rapid development of intelligent algorithms, more and more intelligent algorithms are available. For example, in 2017, Mohamed et al. [9] proposed the moth swarm algorithm; Yang et al. [10] proposed the hunger games search algorithm in 2021; in 2022, Ia et al. [11] proposed the weighted mean of vector algorithm. The equilibrium optimizer (EO) is a new optimization algorithm inspired by the physical phenomenon of control volume mass balance proposed in 2020, and it is characterized by its high optimization finding capability and simple parameters [12], but it still suffers from a tendency to fall into local optima and slow convergence in practical applications. Therefore, it is necessary to improve the algorithm of the equilibrium optimizer to ensure the stability and effectiveness of the algorithm. Wang et al. [13] used backpropagation neural networks to predict more output data, which can achieve more efficient optimization and more reasonable fitness functions; Fan et al. [14] proposed a definition of certain particle concentrations based on OBL, a new nonlinear time control strategy, a novel population update, and a chaos-based strategy. Fu et al. [15] combined the strategies of the modal algorithm and fused EO and heat exchange optimization (TEO) to obtain a new highly equilibrium optimizer (HEO). Gupta et al. [16] used Gaussian variation and an additional exploratory search mechanism based on the concept of population partitioning and reconstruction to improve the convergence speed of the algorithm and obtain more accurate optimal solutions; although these methods achieved good results, the EO algorithm still needs to be improved in terms of convergence speed and accuracy.

This paper proposes a TAFEO algorithm to increase the convergence speed, improve the convergence accuracy, and avoid falling into the local optimum. To verify the effectiveness of the improved algorithm, an LSTM model based on the equilibrium optimizer is then constructed and applied to log reservoir identification to achieve desirable practical application results.

## 2. Materials and Methods

### 2.1. The Equilibrium Optimizer Algorithm and Its Improvements

#### 2.1.1. Equalization Optimizer Algorithm

The equilibrium optimizer (EO) is primarily a physically heuristic optimization algorithm for dynamic mass balance in a strongly mixed type of controlled volume. The mass balance equation embodies the physical processes of mass entry, departure, and generation in the controlled volume and is generally described using a first-order differential equation as shown in

(1) $$\begin{array}{c} V\frac{dC}{dt}=QC_{eq}-QC+G, \end{array}$$

where *V* is the control volume; *C* is the concentration in the control volume; *Q* is the volumetric flow rate into or out of the control volume; *C*_*eq*_ is the concentration within the control volume in the absence of mass production (i.e., at equilibrium); and *G* is the mass production rate within the control volume.

By solving the differential equation described by equation (1), we can find that

(2) $$\begin{array}{c} C=C_{eq}+\left(C_{0}-C_{eq}\right)F+\frac{G\left(1-F\right)}{\lambda V}, \end{array}$$

(3) $$\begin{array}{c} F=\exp\left(-\lambda \left(t-t_{0}\right)\right), \end{array}$$

where *F* is the exponential term factor, *λ* is the flow rate, and *C*_0_ is the initial concentration of the control volume at time *t*_0_.

### 2.2. Improvement of the Equalization Optimizer Algorithm

At present, the equilibrium optimizer algorithm has an excellent performance in intelligent algorithms and has been applied to specific problems with significant effects, but it still suffers from slow convergence speed, insufficient initial population diversity, and ease to fall into local extremes. In this paper, three strategies are proposed to better the equilibrium optimizer algorithm: using tent chaotic mapping to enhance population diversity; introducing convergence factors to accelerate the convergence speed of the algorithm in the early stage, and the ability to search locally in the late stage; and using an early perturbation strategy to enhance the ability of the algorithm to jump out of the local optimum.

#### 2.2.1. Improvement Strategies

*(1) Tent Chaotic Mapping*. The traversal of tent mapping has uniformity and randomness, which enables the algorithm to easily escape from local optimal solutions, thus maintaining the diversity of the population while improving the global search ability. Therefore, to obtain a good initial solution position with a greater chance and speed up the convergence of the population, this paper adopts the tent chaotic mapping method with better traversal uniformity and faster iteration speed to improve the coverage space of the initial solution, which is calculated as shown

(4) $$\begin{array}{c} x\left(t+1\right)=\left\{\begin{array}{cc} \frac{x\left(t\right)}{0.6}, & x\left(t\right)<0.6,\\ \frac{1-x\left(t\right)}{0.4}, & x\left(t\right)\geq 0.6. \end{array}\right. \end{array}$$

Among others, *x*(*t*) ∈ [0,1].

*(2) Convergence Factor*. The exponential term coefficient F used to balance the local search and global search ability in the equalization optimizer algorithm uses constant coefficient weights, and the tendency of the obtained coefficients to change tends to be constant, which does not conform to the nonlinear optimization search law in the algorithm iteration process. To address the problem of slow convergence and low precision in the EO algorithm, a nonlinear decreasing strategy is proposed to balance the local and global search capabilities of the algorithm so that the algorithm has enough steps to search for spatially dispersed populations in the early iterative stage, and reduces the step size to facilitate local search in the late iterative stage of the algorithm, with the convergence factor A defined as shown in

(5) $$\begin{array}{c} A=\frac{2\left[e^{1-{\left(t/t_{\max}\right)}^{2}}-1\right]}{\left(e-1\right)}, \end{array}$$

where *t* is the current number of iterations, and *t*_max_ is the maximum number of iterations.

*(3) Early Perturbation Strategy*. For the disadvantage that the equilibrium optimizer algorithm is easy to fall into the local optimum set, condition to determine whether the particles fall into the local optimum, and update the particle positions when the condition of falling into the local optimum is satisfied, thus introducing the early perturbation strategy, as shown in equations (6) and (7), when equation (12) is satisfied, we reset the positions of the particles so that they are randomly distributed around the gbest thereby jumping out of the local optimum, i.e.,

(6) $$\begin{array}{c} \left|F_{g}\left(t\right)-F_{g}\left(t-1\right)\right|<0.01\cdot \left|F_{g}\left(t\right)\right|, \end{array}$$

(7) $$\begin{array}{c} x\left(t\right)=\left(\text{gbest}\left(t\right)+\text{gbest}\left(t-1\right)\right)\cdot r_{a}, \end{array}$$

where *F*_*g*_(*t*)/*F*_*g*_(*t* − 1) is the value of the function corresponding to the global optimum of the *t*/*t* − 1 generation, respectively, and *r*_*a*_ is a random number of [−1, 1].

#### 2.2.2. Improved Algorithms

Based on the above three improvement strategies, this paper's specific operation process and parameters of the improved algorithm are designed as follows:

(1) *Initialization*. The algorithm performs random initialization within the upper and lower bounds of each optimization variable, as shown in
  (8) $$\begin{array}{c} C_{i}^{0}=C_{\min}+r_{i}\left(C_{\max}-C_{\min}\right),\;i=1,2,\ldots ,n, \end{array}$$
where *C*_min_ and *C*_max_ are the lower and upper bound vectors of the optimization variables, respectively; *r*_*i*_ represents a vector of random numbers for individual *i*, whose dimension is the same as the dimension of the optimization space, with each element value being a random number from 0 to 1.
Using equation (4), the solution is generated by the random initialization.
(2) *Equilibrium State Pool*. To improve the global search capability of the algorithm and avoid falling into low-quality local optimal solutions, the equilibrium state (i.e., the optimal individual) in equation (9) will be selected from within the five currently optimal candidate solutions, which constitute the equilibrium state pool as follows:
  (9) $$\begin{array}{c} C_{eq,\text{pool}}=\left\{C_{eq,1},C_{eq,2},C_{eq,3},C_{eq,4},C_{eq\text{,ave}}\right\}, \end{array}$$
where *C*_*eq*,1_, *C*_*eq*,2_, *C*_*eq*,3_, *C*_*eq*,4_ are the best four solutions found as of the current iteration; *C*_*eq*,ave_ represents the average state of these four solutions. It is worth noting that these five candidate solutions are chosen with the same probability of 0.2.
(3) *Exponential Term CoefficientF*. To better balance the local and global search of the algorithm, equation (3) is improved as follows:
  (10) $$\begin{array}{c} F=a_{1}*\text{sign}\left(r-0.5\right)\left[e^{-\lambda t}-1\right], \end{array}$$
where *a*_1_ is the constant coefficient of the weight of the global search; sign is the symbolic function; and *r*, *λ* all represent vectors of random numbers whose dimensions are the same as the dimension of the optimization space, with each element value being a random number from 0 to 1.
The constant factor weights *a*_1_ are replaced by *A* in equation (5).
(4) *Mass Generation RateG*. To enhance the local search capability of the algorithm, the generation rate is designed as shown in
  (11) $$\begin{array}{c} G=G_{CP}\left(C_{eq}-\lambda C\right), \end{array}$$
  (12) $$\begin{array}{c} G_{CP}=\left\{\begin{array}{cc} 0.5r_{i}, & \text{if }r_{2}\geq 0.5,\\ 0, & \text{otherwise,} \end{array}\right. \end{array}$$
where *G*_*CP*_ is a vector of generation rate control parameters; *r*_*i*_ is a vector of random numbers whose dimension is the same as the dimension of the optimization space, with each element value being a random number from 0 to 1; and *r*_2_ is a random number range from 0 to 1.
(5) *Solution Update*. For optimization problems, the individual solutions can be updated as follows, based on what is shown in

(13) $$\begin{array}{c} C=C_{eq}+\left(C_{0}-C_{eq}\right)F+\frac{G\left(1-F\right)}{\lambda V}. \end{array}$$

We use equation (6) to determine whether it is the optimal solution. If the current output is not the optimal solution, we use equation (7) to reset the particle position.

In summary, the above improvement algorithm that incorporates the three improvement strategies is named TAFEO, and the specific steps of the TAFEO algorithm are shown in Table 1.

### 2.3. Simulation Experiments and Analysis of Results

The computer configuration used for the simulation experiments was Intel Core i7 6700HQ with 3.6 GHz main frequency, 16 GB of memory, 64-bit operating system, and MATLAB R2020b as the computing environment. In the following experiments, we set the number of evaluations to *M* = *N* ∗ *T* = 30000, the number of populations of all intelligent optimization algorithms was set to *N* = 30, and the maximum number of iterations to *T* = 1000. For each basic algorithm, the internal parameter settings are shown in Table 2.

To verify the effectiveness and generalization of the TAFEO algorithm, 16 international standard test functions are used, F1-F10 [17] is chosen from the common test functions, and F11-F16 is from CEC2017 [18]. The details of the test functions are shown in Table 3.

#### 2.3.1. Performance Comparison of Various Improved EO Algorithms

**Figure 1:**
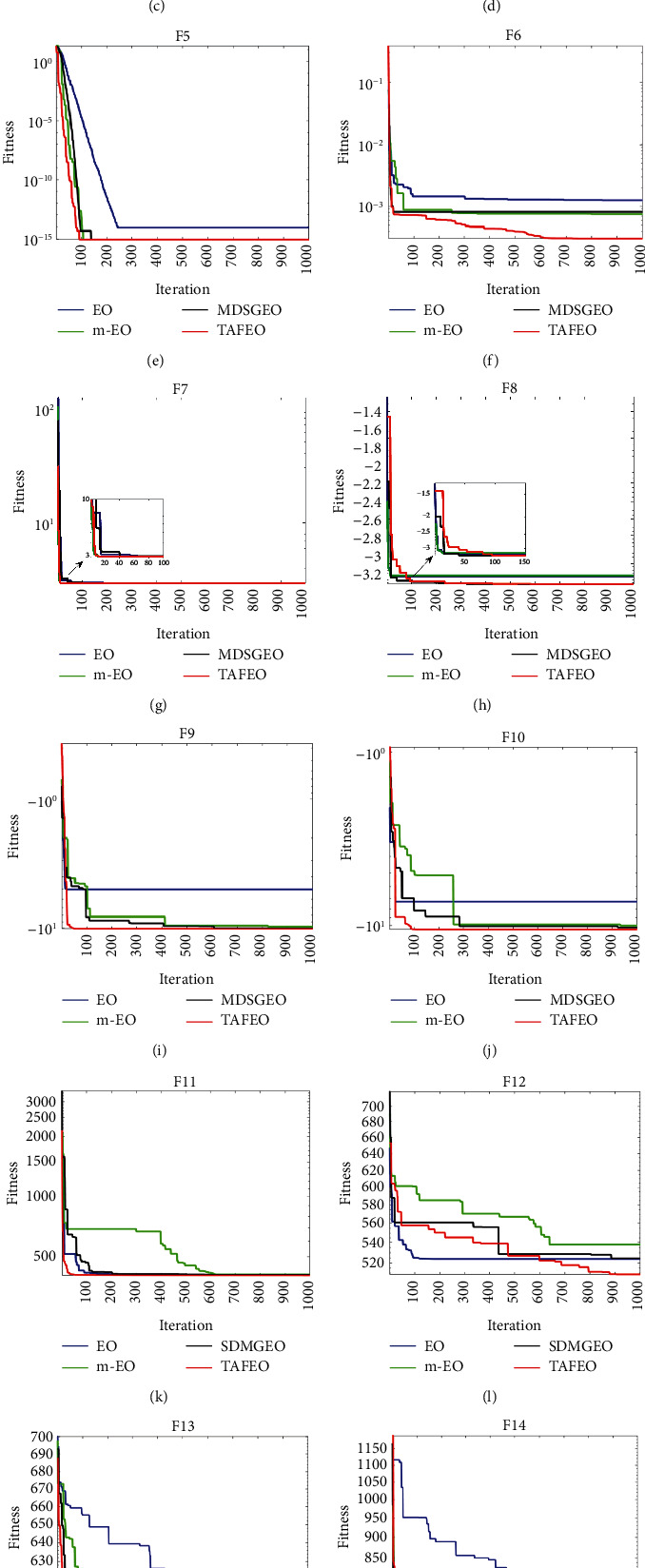
Simulation optimization iteration curve of 16 test benchmark functions. (a) Function F1. (b) Function F2. (c) Function F3. (d) Function F4. (e) Function F5. (f) Function F6. (g) Function F7. (h) Function F8. (i) Function F9. (j) Function F10. (k) Function F11. (l) Function F12. (m)Function F13. (n) Function F14. (o) Function F15. (p) Function F16.

To verify the effectiveness of the improved TAFEO algorithm, the performance of the search iterations was compared with the basic EO algorithm, the m-EO algorithm [16], and the MDSGEO [18] on test functions. Among them, m-EO is proposed in the literature [16] as an improved equilibrium optimizer algorithm using Gaussian variance and based on population partitioning and reconstruction, and the MDSGEO algorithm in literature [18] as an enhanced equilibrium optimizer algorithm using sinusoidal pooling strategy and adaptive preferential gravity strategy. Sixteen benchmark test functions of Table 3 were selected to test the four algorithms. The simulation-optimization-seeking iteration curves are shown in Figure 1.

As shown in Figure 1, the improved TAFEO algorithm performs well in the tests of all the functions, the convergence accuracy is better than all algorithms, and the convergence speed is faster than other algorithms on most test functions except F8, F12, and F14. The comparative analysis shows that the improved strategy of this paper is feasible.

Table 4 shows the results of the different algorithms. To demonstrate the repeatability of each algorithm, the optimal solution and standard deviation in Table 4 are the averages of 30 optimization calculations for each algorithm on sixteen benchmark test functions.

As can be seen from Table 4, the convergence accuracy of the improved TAFEO algorithm is significantly better than that of the original EO algorithm and the two improved EO algorithms in sixteen benchmark functions. The comparative analysis proves that the enhanced TAFEO algorithm in this paper is effective.

#### 2.3.2. Performance Comparison of the Improved EO with Various Intelligent Algorithms

The TAFEO algorithm was compared with the whale algorithm (WOA) [19], the marine predator algorithm (MPA) [20], the wolf pack (GWO) algorithm [21], and the EO algorithm to verify the performance of the TAFEO algorithm.

**Figure 2:**
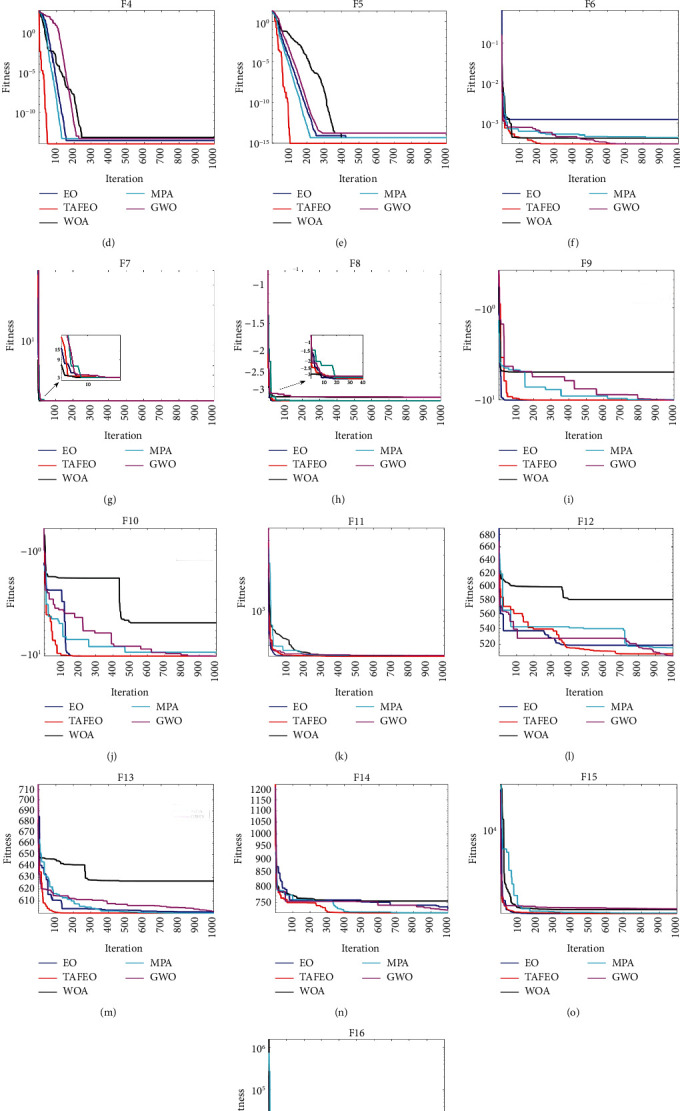
Simulation optimization iteration curve of 16 test benchmark functions. (a) Function F1. (b) Function F2. (c) Function F3. (d) Function F4. (e) Function F5. (f) Function F6. (g) Function F7. (h) Function F8. (i) Function F9. (j) Function F10. (k) Function F11. (l) Function F12. (m) Function F13. (n) Function F14. (o) Function F15. (p) Function F16.

To observe the performance of the five algorithms more clearly, sixteen benchmark test functions were chosen for the iterative graph of the simulation search, and the results are shown in Figure 2.

In Figure 2, the improved TAFEO has the highest convergence accuracy in all test functions except F12 and F16, and the convergence speed is faster than other algorithms in all test functions.  Table 5 shows the results of the different algorithms.

To demonstrate the repeatability of each algorithm, the optimal solution and standard deviation in Table 5 are the averages of 30 optimization calculations for each algorithm on sixteen benchmark test functions.

As can be seen in Table 5, TAFEO outperformed the other four algorithms in terms of both search accuracy and performance stability in the benchmark function test. Therefore, it can be verified that the convergence accuracy and convergence speed of TAFEO are both higher than the other four algorithms.

In summary, the improvements to the equilibrium optimizer algorithm in this paper are highly effective and TAFEO outperforms several other intelligent algorithms tested for the benchmark functions.

#### 2.3.3. Wilcoxon Rank-Sum Test

To observe the statistical difference between the TAFEO algorithm and other algorithms, Wilcoxon rank-sum test[22] was used to verify the results. We compare TAFEO with the EO algorithm, WOA algorithm, MP algorithm, GWO algorithm, m-EO algorithm, and MDSFEO algorithm through 18 test functions in Table 3 to verify the statistical superiority of the TAFEO algorithm. If the *p*-value is greater than 0.05 or NAN, it means that TAFEO is not statistically significantly different on this function. The Wilcoxon rank-sum test results are shown in Table 6.

The bold text in Table 6 indicates values greater than 5% or NAN. From Table 6, on F1, the result of the MDSGEO test is NAN, because both TAFEO and MDSGEO seek the best theoretical solution at the same time, so there is no statistical difference. There was no significant difference on F7 between TAFEO and m-EO. There was no significant difference between TAFEO and MPA on F7 and F15. There was no significant difference between TAFEO and GWO on F12 and F16.

In addition to the above, there are statistical differences between the TAFEO and other algorithms in the Wilcoxon rank-sum test, which shows that the TAFEO algorithm has great statistical advantages in the optimization results of benchmark function and verifies the robustness of the algorithm.

## 3. Improvements to the LSTM

### 3.1. LSTM Principle

**Figure 3:**
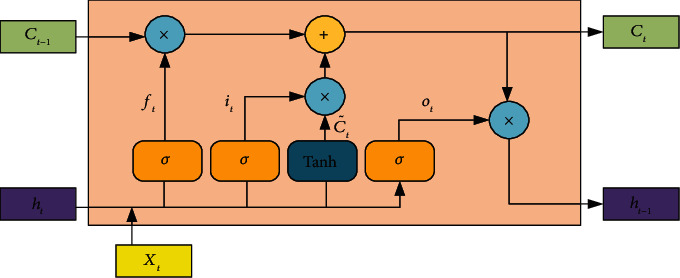
Cell unit structure of LSTM.

The long short-term memory (LSTM) network is a recurrent neural network responsible for computing the dependencies between observations in a time series. As such, it is commonly used for forecasting. Because logging attribute data are time-series data, this paper uses LSTM as a classification prediction model for reservoir identification. The cell structure of the primary LSTM neural network is shown in Figure 3.

An LSTM cell element includes the forgetting gate *f*_*t*_, the input gate *i*_*t*_, and the output gate *o*_*t*_, which are used to protect and control.

Information *i*_*t*_ determines the new information being stored in the cell state, ${\tilde{C}}_{t}$ is used to determine the updated information, and finally, the *o*_*t*_ gate determines the output value to the next LSTM cell element.

The equation for each variable in the LSTM network is shown in

(14) $$\begin{array}{c} f_{t}=\sigma \left(W_{f}\left[h_{t-1},x_{t}\right]+b_{f}\right),\\ i_{t}=\sigma \left(W_{i}\left[h_{t-1},x_{t}\right]+b_{i}\right),\\ o_{t}=\sigma \left(W_{o}\left[h_{t-1},x_{t}\right]+b_{o}\right),\\ {\tilde{C}}_{t}=\tanh\left(W_{c}\left[h_{t-1},x_{t}\right]+b_{c}\right),\\ C_{t}=f_{t}C_{t-1}+{\tilde{C}}_{t},\\ h_{t}=O_{t}\tanh\left(C_{t}\right), \end{array}$$

where *W*_*f*_, *W*_*i*_, *W*_*o*_, *W*_*c*_ are the weight matrix; *b*_*f*_, *b*_*i*_, *b*_*o*_, *b*_*c*_ are the offset vector; *σ* is the sigmoid activation function and takes values in the range [0, 1]; and tanh is the tangent activation function and takes values in the range [−1, 1].

### 3.2. Research on the TAFEO-LSTM Model

#### 3.2.1. Improvement Strategies

In the basic LSTM neural network, the number of neurons in the hidden layer is mainly chosen randomly or empirically, which leads to the problems of low classification accuracy and unstable classification effect. When the number of Batchsize is too large, it will increase the memory capacity and make the gradient descent direction no longer change, which will easily fall into the local optimal solution and reduce the accuracy. The size of the Maxepoch determines whether the model can be fitted or not; when the Maxepoch is too small, the model will be underfitted, and when the Maxepoch is too large, the model will be overfitted. Therefore, the TAFEO algorithm can be used to optimize the hyperparameters and the number of neurons of the LSTM network to enhance the classification accuracy and speed up the classification. In this paper, TAFEO is combined with the LSTM network to optimize the number of neurons in the hidden layer and the parameters batchsize and maxepoch in gradient descent of the LSTM model.

**Figure 4:**
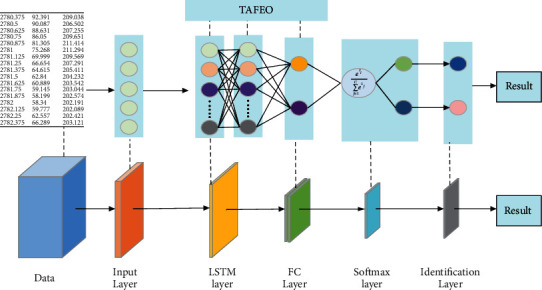
Structure of the TAFEO-LSTM model.

The improved TAFEO-LSTM model is shown in Figure 4.

The algorithm table for the improved TAFEO-LSTM model is shown in Table 7.

### 3.3. UCI Dataset Simulation Experiments

To test the superiority of the improved algorithm optimization model, six sets of international general UCI binary classification datasets were selected for comparison. The comparison models include LSTM, EO-LSTM, and TAFEO-LSTM, and the six datasets are banknote, blood, climate simulation, Indian, Pima, and WDBC. The details of the six datasets are shown in Table 8.

To evaluate the performance of the model more accurately, the ROC [23] curve was introduced as an additional metric for model evaluation in addition to the accuracy.

The vertical coordinate of the ROC curve is the true-positive rate (TPR), and the horizontal coordinate is the false-positive rate (FPR). The true-positive rate represents the proportion of predicted positive samples to all positive samples, as shown in equation (15), and the false-positive rate represents the proportion of predicted positive samples to all negative samples, as shown in

(15) $$\begin{array}{c} \text{TPR}=\frac{TP}{\left(TP+FN\right)}, \end{array}$$

where *TP* is the number of positive examples of correctly classified labels, and FN is the number of negative examples of incorrectly classified labels.

(16) $$\begin{array}{c} \text{FPR}=\frac{FP}{\left(FP+TN\right)}, \end{array}$$

where TN is the number of negative examples of correctly classified labels, and FP is the number of positive examples of incorrectly classified labels.

In the ROC curve, the performance of the model is usually evaluated by the value of AUC (area under ROC curve), which is the area under the ROC curve, and the larger the AUC value, the better the generalization performance of the model. The formula for calculating AUC is shown in

(17) $$\begin{array}{c} \text{AUC}=\frac{\sum_{i\in \text{positiveclass}}{\text{rank}}_{i}-\left(M\left(1+M\right)/2\right)}{M\times N}, \end{array}$$

where *M* and *N* are the number of positive and negative samples, respectively.

**Figure 5:**
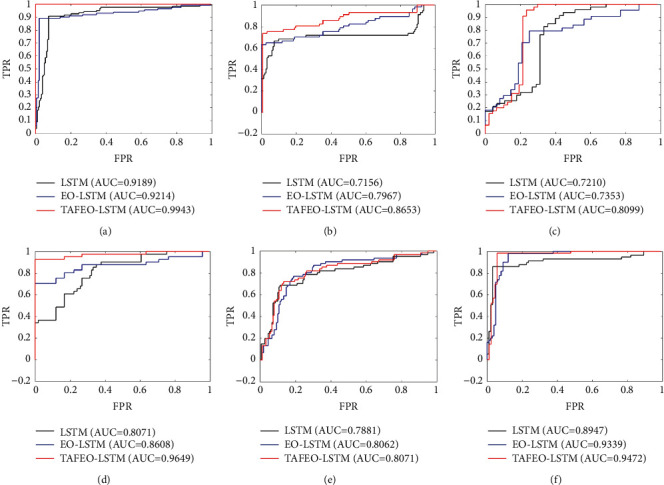
ROC curves of six groups of UCI test sets. (a) Banknote. (b) Blood. (c) Climate simulation. (d) Indian. (e) Pima. (f) WDBC.

In the simulation experiments, each UCI dataset was divided into a 70% training set and a 30% test set. The experimental results were averaged over ten experiments. The AUC values of classification results are shown in Table 9, and the ROC prediction curves are shown in Figure 5.

From Figure 5, it can be seen that the strategy of using TAFEO for the LSTM model with the number of neurons seeking and hyperparameters batchsize and maxepoch seeking is effective, and the AUC value of LSTM with the original LSTM model has been improved than after EO optimization. As can be seen from Table 8, the accuracy of the LSTM model optimized by TAFEO is also improved.

## 4. Logging Reservoir Identification

### 4.1. Logging Dataset

To verify the effectiveness of the TAFEO-LSTM model proposed in this paper in oil logging data mining, actual oil and gas field data (D1 and D2) were used for validation.

**Figure 6:**
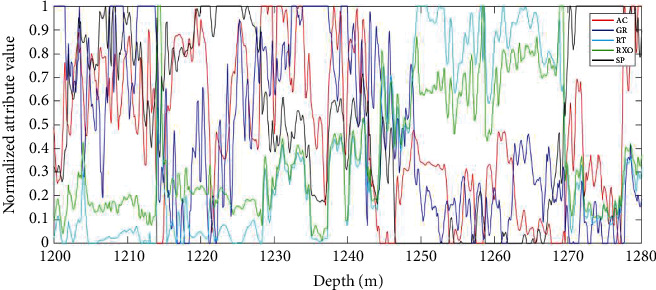
Attribute normalization graph of D1.

**Figure 7:**
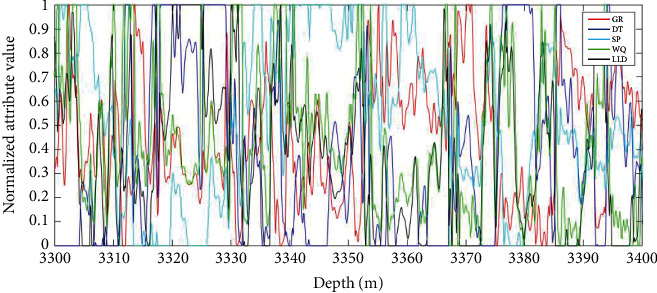
Attribute normalization graph of D2.

The D1 well was attribute reduced to obtain five attributes, namely, (AC, GR, RT, RXO, and SP), and the D2 well was attribute reduced to obtain 13 attributes, namely, (GR, DT, SP, WQ, LLD, LLS, DEN, NPHI, PE, U, TH, K, and CALI). The attributes of D1 and D2 wells were applied in the testing process after attribute simplification, and the data in the training well segment were divided into 70% training set and 30% test set. The attribute information from the D1 and D2 wells was selected for normalization, and the five main attributes from each of the D1 and D2 wells were selected to draw their logging curves, as shown in Figures 6 and 7.

To verify the performance of the TAFEO-LSTM model, five models, LSTM, EO-LSTM, MPA-LSTM, WOA-LSTM, and GWO-LSTM, are constructed for comparison experiments. The parameter settings in each model are shown in Table 10.

Information on the data from the two selected wells is shown in Table 11.

### 4.2. Reservoir Identification Results

**Figure 8:**
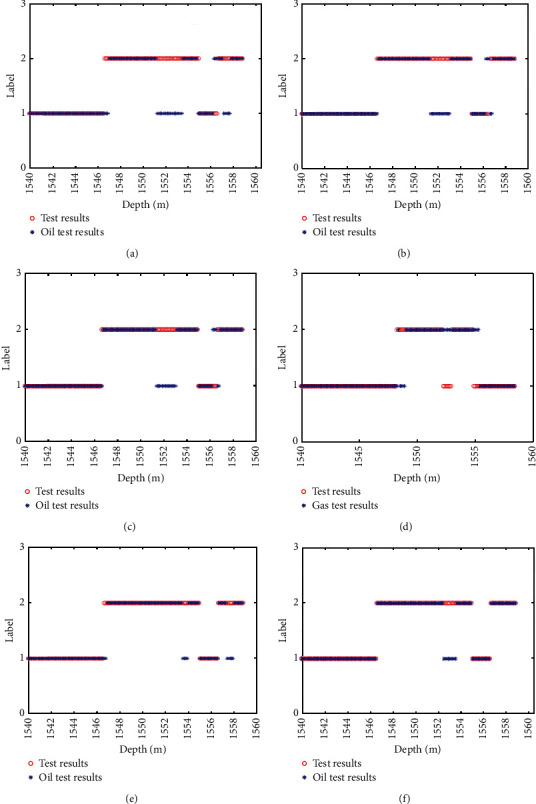
The actual oil layer distribution of D1 and classification of LSTM. (a) Classification of LSTM. (b) Classification of MPA-LSTM. (c) Classification of WOA-LSTM. (d) Classification of GWO-LSTM. (e) Classification of EO-LSTM. (f) Classification of TAFEO-LSTM.

Figure 8 shows the actual formation results for each algorithm model in the test well section of well D1 compared to the test oil results, where vertical coordinate 2 represents the oil formation and vertical coordinate 1 represents the nonoil formation.

From Figure 8, the TAFEO-LSTM model has higher accuracy and the classification results can be closer to the actual oil layer distribution than the LSTM, EO-LSTM, MPA-LSTM, WOA-LSTM, and GWO-LSTM, and the accuracy was used to assess the model performance. The recognition accuracy was selected as the average value of each algorithm after 30 runs. The results are shown in Table 12.

From Table 11, the classification performance of the LSTM optimized by applying the intelligent algorithm has significantly improved, in which the recognition accuracy of the oil layer can reach 94.04% by applying the TAFEO-LSTM model, which indicates that it is feasible to apply TAFEO to optimize the LSTM and identify the oil layer with significant effect.

Similar to D1, accuracy was used to assess model performance and the average of results after 30 runs is shown in Table 13.

From Table 13, the classification performance of the LSTM optimized by applying the intelligent algorithm is significantly improved, in which the recognition accuracy of the gas layer can reach 95.01% by applying the TAFEO-LSTM model, which indicates that it is feasible to apply the TAFEO-optimized LSTM and the recognition of the gas layer is significant.

**Figure 9:**
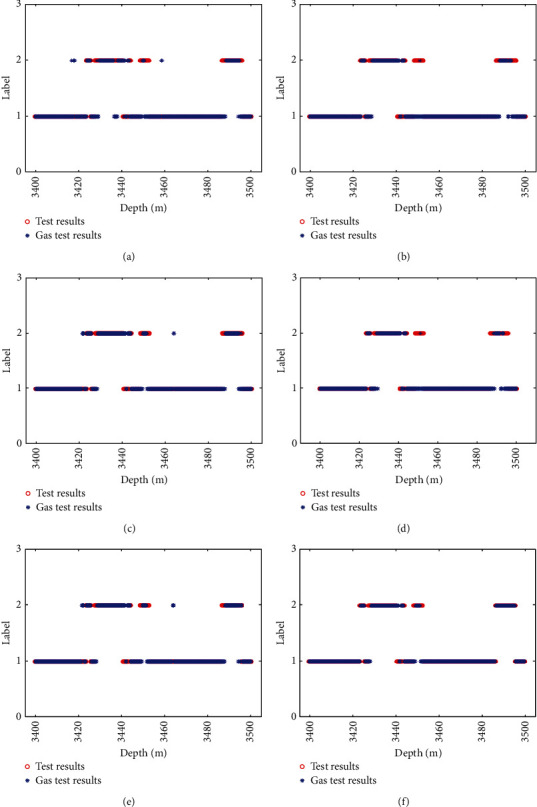
The actual gas layer distribution of D2 and classification of LSTM. (a) Classification of LSTM. (b) Classification of MPA-LSTM. (c) Classification of WOA-LSTM. (d) Classification of GWO-LSTM. (e) Classification of EO-LSTM. (f) Classification of TAFEO-LSTM.

Figure 9 shows the actual gas formation results for each algorithm model in the test well section of well D2 compared to the test gas results, where vertical coordinate 2 represents the gas formation and vertical coordinate 1 represents the nongas formation.

As can be seen from Figure 9, compared with LSTM, EO-LSTM, WOA-LSTM, GWO-LSTM, and MPA-LSTM, the TAFEO-LSTM model has a higher accuracy rate and the classification results can be closer to the real gas layer distribution.

### 4.3. Comparison of Reservoir Identification Models

To better verify the validity of the improved model, several models that have been applied to logging are compared with those proposed in this paper. These models are Fisher's different approach, BP neural network, ELM [24], SVM [25], and CNN [26]. We use these models to identify well D2 logging data. The comparison results are shown in Table 14.

From Table 14, the TAFEO-LSTM model is more accurate in identifying reservoirs than other models because it takes into account the temporal characteristics of logging data.

In summary, the improved TAFEO strategy applying LSTM classification is effective in practical logging reservoir identification.

## 5. Conclusions

1. An improved equilibrium optimizer algorithm, TAFEO, is proposed, and tent mapping is introduced to increase the population diversity. The introduction of a convergence factor is to effectively accelerate the convergence speed of the algorithm and balance the local and global optimization-seeking ability, and finally, adding a premature perturbation strategy can prevent the algorithm from falling into the local optimum. Through the simulation of 16 benchmark functions and Wilcoxon rank-sum test for optimization results, the improved algorithm outperforms various intelligent optimization algorithms in terms of accuracy and convergence speed and has good robustness.
2. The TAFEO algorithm is applied to the LSTM parameter optimization; then, a reservoir identification TAFEO-LSTM model is established. Simulation experiments on the UCI dataset demonstrate that the improved model has a strong generalization ability and high recognition rate. The TAFEO-LSTM model is then applied to identify the reservoirs, and the results are compared with those of five reservoir identification models, namely, LSTM, EO-LSTM, WOA-LSTM, GWO-LSTM, and MPA-LSTM. The results show that the proposed TAFEO-LSTM model is more accurate than the other five models in identifying reservoirs, with the highest accuracy of 94.04% for the oil layer and 95.01% for the gas layer. Subsequently, TAFEO-LSTM still performs well compared with the other models used for reservoir identification. Obviously, the improved model is effective in reservoir identification and has broad application prospects.

## Figures and Tables

**Table 1:** Algorithm steps of TAFEO.

Algorithm: TAFEO

Input: Population size: *N*, the maximum number of iterations: *T*, number of dimensions: *D*, parameters: *a*_1_, *a*_2_, GP
Output: Global optimal position *g*_best_ and global optimal position adaptation *f*_*g*_best__
1. Initializing the location of the population using the tent chaotic mapping
2. Set up four initial equilibrium candidate solutions
3. While *t* < *T*
4. For *i* = *i*: D
5. Calculate the fitness of the four equilibrium candidate solutions
6. $\text{find}{\overset{⟶}{C}}_{eq1}∼{\overset{⟶}{C}}_{eq4}$
7. End for
8. For *i* = *i*: N
9: Find the best fit by comparing the fit between particles
10: End
11: Calculation ${\overset{⟶}{C}}_{\text{ave}}$
12: Building a balanced pool
13: For *i* = *i*: N
14: Generate the exponential term coefficients *F*, the mass generation rate *G* and calculate the optimal solution according to equations (5), (10)–(12) and according to equation (13)
15. Introduce a premature perturbation strategy to determine whether to fall into a local optimum solution and to determine whether to reset the particle position
16: End
17: Update the global optimal position *g*_best_ and the global optimal position adaptation *f*_*g*_*best*__
18: *t* = *t*+1
19: End while

**Table 2:** Parameter settings of the optimization algorithms.

Algorithms	Parameter settings
EO	*a*_1_ = 2, *a*_2_ = 1, *GP* = 0.5
m-EO	*a*_1_ = 2, *a*_2_ = 1, *GP* = 0.5
MDSGEO	*a*_1_ = 2, *a*_2_ = 1, *GP* = 0.5
TAFEO	*a*_1_ = 2, *a*_2_ = 1, *GP* = 0.5
WOA	*a* ∈ [2,0], *b* = 1, *l* ∈ [−1,1]
GWO	*a* ∈ [2,0]
MPA	FAD_*s*_ = 0.2, *P* = 0.5

**Table 3:** Sixteen benchmark functions.

Function	Dim	Range	Optimal value
*F*_1_(*x*) = ∑_*i*=1_^*n*^|*x*_*i*_| + ∏_*i*=1_^*n*^|*x*_*i*_|	30	[−10,10]	0
*F*_2_(*x*) = ∑_*i*=1_^*n*^[*x*_*i*_ + 0.5]	30	[−100, 100]	0
*F*_3_(*x*) = ∑_*i*=1_^*n*^*ix*_*i*_^4^ + random[0,1)	30	[−1.28, 1.28]	0
*F*_4_(*x*) = ∑_*i*=1_^*n*^[*x*_*i*_^2^ − 10cos(2*πx*_*i*_) + 10]	30	[−500, 500]	0
$F_{5}\left(x\right)=-20\exp\left(-0.2\sqrt{1/n\sum_{i=1}^{n}x_{i}^{2}}\right)-\exp\left(1/n\sum_{i=1}^{n}\cos\left(2\pi x_{i}\right)\right)+20+e$	30	[−5.12, 5.12]	0
*F*_6_(*x*) = ∑_*i*=1_^11^[*a*_*i*_ − *x*_1_(*b*_*i*_^2^ + *b*_*i*_*x*_2_)/*b*_*i*_^2^+*b*_*i*_*x*_3_+*x*_4_]^2^	4	[−5, 5]	0.00030
$\begin{array}{c} F_{7}\left(x\right)=\left[1+{\left(x_{1}+x_{2}+1\right)}^{2}\left(19-14x_{1}+3x_{1}^{2}-14x_{2}+6x_{1}x_{2}+3x_{2}^{2}\right)\right]\times \\ \left[30+{\left(2x_{1}-3x_{2}\right)}^{2}\times \left(18-32x_{1}+12x_{1}^{2}+48x_{2}-36x_{1}x_{2}+27x_{2}^{2}\right)\right] \end{array}$	2	[−2, 2]	3
*F*_8_(*x*) = −∑_*i*=1_^4^*c*_*i*_exp(∑_*j*=1_^6^*a*_*ij*_(*x*_*j*_ − *p*_*ij*_)^2^)	6	[0, 1]	−3.32
*F*_9_(*x*) = −∑_*i*=1_^5^[(*X* − *a*_*i*_)(*X* − *a*_*i*_)^*I*^ + *c*_*i*_]^−1^	4	[0, 10]	−10.1532
*F*_10_(*x*) = −∑_*i*=1_^10^[(*X* − *a*_*i*_)(*X* − *a*_*i*_)^*T*^ + *c*_*i*_]^−1^	4	[0, 10]	−10.5363
$\begin{array}{c} F_{11}\left(x\right)=f\left(M\left(2.048\left(x-o_{4}\right)/100\right)+1\right)+{F_{11}}^{*}\\ f\left(x\right)=\sum_{i=1}^{D-1}\left(100{\left(x_{i}^{2}-x_{i+1}\right)}^{2}+{\left(x_{i}-1\right)}^{2}\right) \end{array}$	30	[−100, 100]	400
$\begin{array}{c} F_{12}\left(x\right)=f\left(M\left(|x-o_{5}\right)\right)+{F_{12}}^{*}\\ f\left(x\right)=\sum_{i=1}^{D-1}\left(x_{i}^{2}-10\cos\left(2\pi x_{i}\right)+10\right) \end{array}$	30	[−100, 100]	500
$\begin{array}{c} F_{13}\left(x\right)=f\left(M\left(0.5\left(x-o_{6}\right)/|100\right)\right)+{F_{13}}^{*}\\ f\left(x\right)=g\left(x_{1},x_{2}\right)+g\left(x_{2},x_{3}\right)+\cdots +g\left(x_{D-1},x_{D}\right)+g\left(x_{D},x_{1}\right)\\ g\left(x,y\right)=0.5+\left(\sin^{2}\left(\sqrt{x^{2}+y^{2}}\right)-0.5\right)/{\left(1+0.001\left(x^{2}+y^{2}\right)\right)}^{2} \end{array}$	30	[−100, 100]	600
$\begin{array}{c} F_{14}\left(x\right)=f\left(M\left(600\left(x-o_{7}\right)/100\right)\right)+{F_{14}}^{*}\\ f\left(x\right)=\min\left(\sum_{i=1}^{D-1}{\left({\hat{x}}_{i}-\mu_{0}\right)}^{2},dD+s\sum_{i=1}^{D-1}{\left({\hat{x}}_{i}-\mu_{1}\right)}^{2}\right)+10\left(D-\sum_{i=1}^{D-1}\cos\left(2\pi {\hat{z}}_{i}\right)\right)\\ \mu_{0}=2.5,\mu_{1}=-\sqrt{\mu_{0}^{2}-d/s},s=1-1/2\sqrt{D+20}-8.2,d=1\\ y=10\left(x-o\right)/100,{\hat{x}}_{i}=2\text{sign}\left(x_{i}^{*}\right)y_{i}+\mu_{0},\;\text{for }i=1,2,\ldots ,D\\ z=\Lambda^{100}\left(\hat{x}-\mu_{0}\right) \end{array}$	30	[−100, 100]	700
F15 composition function 1	30	[−100, 100]	1100
*N* = 3 *p* = [0.2, 0.4, 0.4]			
*g*_1_: *Zakharov function*			
*g*_2_: *Rosenbrock function*			
*g*_3_: *Rastrigin's function*			
F16 composition function 2	30	[−100, 100]	1400
*N* = 4 *p* = [0.2, 0.2, 0.4, 0.4]			
*g*_1_: *High Conditioned elliptic function*			
*g*_2_: *Ackley's function*			
*g*_3_: *Schaffer's F7 function*			
*g*_4_: *Rastrigin's function*			

**Table 4:** Test results of different algorithms.

Functions	Algorithms	Optimum solution	Standard deviation
F1	EO	4.950*e* − 48	8.168*e* − 48
	m-EO	3.911–315	1.256*e* − 314
	MDSGEO	0.000 + 00	0.000*e* + 00
	TAFEO	0.000 + 00	0.000*e* + 00

F2	EO	4.205*e* + 00	3.730*e* − 01
	m-EO	3.492*e* − 01	1.435*e* − 01
	MDSGEO	2.752–01	2.015–01
	TAFEO	3.293*e* − 09	9.608–09

F3	EO	5.467*e* − 04	2.592*e* − 03
	m-EO	6.068*e* − 05	5.926*e* − 05
	MDSGEO	5.395*e* − 05	4.392*e* − 05
	TAFEO	4.843*e* − 05	2.123*e* − 05

F4	EO	5.684*e* − 14	1.257*e* − 14
	m-EO	1.023*e* − 12	3.854*e* − 12
	MDSGEO	7.031*e* − 11	6.255*e* − 12
	TAFEO	3.215*e* − 14	5.665*e* − 15

F5	EO	5.388*e* − 15	1.597*e* − 15
	m-EO	9.613*e* − 16	3.236*e* − 16
	MDSGEO	8.881*e* − 16	7.556*e* − 15
	TAFEO	8.123*e* − 16	0.000*e* + 00

F6	EO	3.082*e* − 03	1.439*e* − 05
	m-EO	3.322*e* − 03	2.596*e* − 02
	MDSGEO	2.342*e* − 04	1.183*e* − 02
	TAFEO	1.235*e* − 05	1.233*e* − 07

F7	EO	3.000*e* + 00	0.000*e* + 00
	m-EO	3.000*e* + 00	0.000*e* + 00
	MDSGEO	3.001*e* + 00	3.694*e* − 03
	TAFEO	3.000*e* + 00	0.000*e* + 00

F8	EO	−3.264*e* + 00	6.378*e* − 02
	m-EO	−3.171*e* + 00	7.977*e* − 02
	MDSGEO	−3.296*e* + 00	4.939*e* − 02
	TAFEO	−3.304*e* + 00	4.342*e* − 02

F9	EO	−4.910*e* + 00	7.650*e* − 01
	m-EO	−8.793*e* + 00	2.292*e* + 00
	MDSGEO	−9.555*e* + 00	5.905*e* − 01
	TAFEO	−9.581*e* + 00	5.871*e* − 01

F10	EO	−5.077*e* + 00	9.873*e* − 01
	m-EO	−10.08*e* + 00	7.358*e* − 01
	MDSGEO	−10.15*e* + 00	6.072*e* − 01
	TAFEO	−10.35*e* + 00	3.018*e* − 01

F11	EO	4.231*e* + 02	2.523*e* + 01
	m-EO	4.143*e* + 02	3.311*e* + 01
	MDSGEO	4.169*e* + 02	2.202*e* + 01
	TAFEO	4.046*e* + 02	1.648*e* + 00

F12	EO	5.182*e* + 02	5.445*e* + 01
	m-EO	5.384*e* + 02	6.240*e* + 01
	MDSGEO	5.318*e* + 02	6.654*e* + 01
	TAFEO	5.121*e* + 02	4.701*e* + 01

F13	EO	6.036*e* + 02	1.105*e* + 00
	m-EO	6.006*e* + 02	2.452*e* + 00
	MDSGEO	6.021*e* + 02	7.086*e* − 01
	TAFEO	6.000*e* + 02	2.986*e* − 04

F14	EO	7.323*e* + 02	6.890*e* + 00
	m-EO	7.490*e* + 02	6.892*e* + 00
	MDSGEO	7.441*e* + 02	7.576*e* + 00
	TAFEO	7.242*e* + 02	6.527*e* + 00

F15	EO	1.319*e* + 03	1.431*e* + 02
	m-EO	1.155*e* + 03	8.634*e* + 01
	MDSGEO	1.161*e* + 03	7.213*e* + 01
	TAFEO	1.109*e* + 03	6.124*e* + 00

F16	EO	1.518*e* + 03	4.208*e* + 01
	m-EO	1.623*e* + 03	1.075*e* + 02
	MDSGEO	1.625*e* + 03	1.482*e* + 02
	TAFEO	1.449*e* + 03	1.829*e* + 01

**Table 5:** Test results of different algorithms.

Functions	Algorithms	Optimum solution	Standard deviation
F1	EO	2.372*e* − 48	4.099*e* − 48
	WOA	1.467*e* − 101	7.826*e* − 101
	GWO	1.830*e* − 33	1.377*e* − 33
	MPA	6.855*e* − 27	1.199*e* − 26
	TAFEO	**4.129*e* − 309**	**0.000*e* + 00**

F2	EO	2.192*e* + 00	1.504*e* + 00
	WOA	5.741*e* − 02	7.447*e* − 02
	GWO	5.227*e* − 01	7.467*e* − 03
	MPA	1.984*e* − 09	1.693*e* − 09
	TAFEO	**9.569*e* − 10**	**9.929*e* − 10**

F3	EO	6.115*e* − 04	3.729*e* − 04
	WOA	2.475*e* − 03	3.002*e* − 03
	GWO	7.951*e* − 04	5.157*e* − 04
	MPA	9.046*e* − 04	4.133*e* − 04
	TAFEO	**4.952*e* − 05**	**4.662*e* − 05**

F4	EO	3.215*e* − 14	2.321*e* − 15
	WOA	7.684*e* − 14	5.288*e* − 14
	GWO	5.625*e* − 14	1.734*e* − 14
	MPA	5.684*e* − 14	3.131*e* − 15
	TAFEO	**1.241 ** *e* − **14**	**8.654** *e* − **16**

F5	EO	5.033*e* − 15	1.346*e* − 15
	WOA	4.204*e* − 15	2.627*e* − 15
	GWO	1.604*e* − 14	4.268*e* − 15
	MPA	4.204*e* − 15	9.013*e* − 16
	TAFEO	**8.881*e* − 16**	**1.256*e* − 16**

F6	EO	3.082*e* − 04	1.439*e* − 06
	WOA	3326*e* − 03	1.821*e* − 02
	GWO	5.034*e* − 03	8.604*e* − 03
	MPA	7.443*e* − 04	3.619*e* − 04
	TAFEO	**3.075*e* − 04**	**2.965*e* − 19**

F7	EO	**3.000*e* + 00**	**0.000*e* + 00**
	WOA	**3.001*e* + 00**	3.731*e* − 05
	GWO	**3.001*e* + 00**	9.491*e* − 14
	MPA	**3.000*e* + 00**	**0.000*e* + 00**
	TAFEO	**3.000*e* + 00**	**0.000*e* + 00**

F8	EO	−3.252*e* + 00	6.920*e* − 02
	WOA	−3.206*e* + 00	1.868*e* − 01
	GWO	−3.263*e* + 00	7.182*e* − 02
	MPA	−3.266*e* + 00	7.754*e* − 02
	TAFEO	−**3.321*e* + 00**	**1.529*e* − 15**

F9	EO	−8.628*e* + 00	2.369*e* + 00
	WOA	−8.128*e* + 00	2.990*e* + 00
	GWO	−9.646*e* + 00	1.546*e* + 00
	MPA	−9.651*e* + 00	5.676*e* − 01
	TAFEO	−**1.153*e* + 01**	**5.760*e* − 15**

F10	EO	−9.905*e* + 00	1.968*e* + 00
	WOA	−9.587*e* + 00	2.156*e* + 00
	GWO	−9.995*e* + 00	2.058*e* + 00
	MPA	−1.018*e* + 01	3.707*e* − 01
	TAFEO	−**1.015***e* + **01**	**1.581** *e* − **15**

F11	EO	4.041*e* + 02	1.923*e* + 00
	WOA	4.149*e* + 02	2.371*e* + 01
	GWO	4.413*e* + 02	5.487*e* + 01
	MPA	4.198*e* + 02	2.541*e* + 02
	TAFEO	**4.000** *e* + **02**	**1.599** *e* − **02**

F12	EO	5.113*e* + 02	4.856*e* + 00
	WOA	5.165*e* + 02	4.753*e* + 00
	GWO	5.551*e* + 02	2.008*e* + 01
	MPA	**5.095** *e* + **02**	7.990*e* + 00
	TAFEO	5.129*e* + 02	**3.401** *e* + **00**

F13	EO	6.015*e* + 02	2.472*e* + 00
	WOA	6.005*e* + 02	1.591*e* − 01
	GWO	6.356*e* + 02	1.278*e* − 01
	MPA	6.000*e* + 02	**4.117** *e* − **03**
	TAFEO	**6.001** *e* + **02**	4.577*e* − 01

F14	EO	7.329*e* + 02	1.197*e* + 01
	WOA	7.317*e* + 02	6.667*e* + 00
	GWO	7.880*e* + 02	2.475*e* + 01
	MPA	7.228*e* + 02	**4.043** *e* + **00**
	TAFEO	**7.217** *e* + **02**	6.230*e* + 00

F15	EO	1.106*e* + 03	3.223*e* + 00
	WOA	1.169*e* + 03	1.028*e* + 00
	GWO	1.225*e* + 03	6.739*e* + 01
	MPA	1.102*e* + 03	5.712*e* + 01
	TAFEO	**1.147** *e* + **03**	**1.28** *e* + **00**

F16	EO	1.460*e* + 03	3.483*e* + 01
	WOA	2.442*e* + 03	4.952*e* + 03
	GWO	**1.403** *e* + **03**	7.159*e* + 01
	MPA	2.411*e* + 03	1.583*e* + 03
	TAFEO	1.448*e* + 03	**2.150** *e* + **00**

**Table 6:** Wilcoxon rank-sum test results.

Function	*P*_*EO*_	*P*_WOA_	*P*_MPA_	*P*_GWO_	*P*_*m*−*EO*_	*P*_MDSGEO_
F1	1.212*e* − 12	1.212*e* − 12	1.212*e* − 12	1.212*e* − 12	1.212*e* − 12	**NAN**
F2	1.212*e* − 12	1.212*e* − 12	1.212*e* − 12	1.212*e* − 12	1.212*e* − 12	1.212*e* − 12
F3	1.212*e* − 12	1.212*e* − 12	1.212*e* − 12	1.212*e* − 12	1.212*e* − 12	1.212*e* − 12
F4	1.212*e* − 12	1.212*e* − 12	1.212*e* − 12	1.212*e* − 12	1.212*e* − 12	1.212*e* − 12
F5	3.020*e* − 11	3.368*e* − 05	3.020*e* − 11	6.414*e* − 1	3.020*e* − 11	3.020*e* − 11
F6	7.389*e* − 11	1.856*e* − 09	2.609*e* − 10	6.695*e* − 11	3.019*e* − 11	9.918*e* − 11
F7	**NAN**	1.608*e* − 13	**NAN**	1.351*e* − 13	**NAN**	6.324*e* − 03
F8	2.708*e* − 14	1.997*e* − 11	3.941*e* − 12	1.195*e* − 13	3.941*e* − 12	3.941*e* − 12
F9	4.113*e* − 11	7.226*e* − 11	4.193*e* − 02	4.193*e* − 02	5.327*e* − 09	8.223*e* − 03
F10	1.193*e* − 06	2.068*e* − 02	2.945*e* − 11	2.773*e* − 05	2.945*e* − 11	2.945*e* − 11
F11	4.077*e* − 11	3.020*e* − 11	3.020*e* − 11	3.020*e* − 11	3.020*e* − 11	3.020*e* − 11
F12	2.010*e* − 11	3.020*e* − 11	3.182*e* − 04	**2.196*e* − 01**	3.020*e* − 11	3.690*e* − 11
F13	5.570*e* − 10	3.020*e* − 11	6.548*e* − 04	3.157*e* − 10	4.074*e* − 11	2.370*e* − 10
F14	4.801*e* − 07	4.203*e* − 11	1.907*e* − 02	1.325*e* − 04	3.690*e* − 11	7.389*e* − 11
F15	1.067*e* − 07	3.005*e* − 03	**4.077*e* − 01**	2.068*e* − 02	2.377*e* − 07	1.173*e* − 03
F16	8.484*e* − 09	3.020*e* − 11	8.684*e* − 03	**5.692*e* − 01**	1.040*e* − 04	2.157*e* − 03

**Table 7:** Algorithm steps of the TAFEO-LSTM model.

Algorithm: the TAFEO-LSTM model algorithm
Inputs: Training set, test set, parameters of TAFEO
Output: TAFEO-LSTM model, test set labels, accuracy
1. Parameters for initializing the LSTM
2. Normalized data processing
3. Initializing the population
4. Calculate the fitness function *f*_gbest_ = 1 − 1/*N*∑_*i*=1_^*N*^(*Y*_*i*_ = *T*_*i*_) and find the current optimal solution
5. While *t* < *T*
6. For *i* = *i*: *N*
7. Determine particle state and update particle position
8. End for
9. Updating the TAFEO-LSTM models to predict classification accuracy
10. *t* = *t* + 1
11. End while
12. The optimal number of neurons with the hyperparameters batchsize and maxepoch is given to the TAFEO-LSTM model for retraining.
13. Building a TAFEO-LSTM model
14. Predictive classification of the test set
15. Calculate classification accuracy, AUC area, and draw ROC curves

**Table 8:** Datasets of UCI.

Datasets	Sample size	Number of features	Training set	Test set
Banknote	1000	4	700	300
Blood	700	4	490	210
Climate simulation	500	18	350	150
Indian	500	8	350	150
Pima	700	8	490	210
WDBC	500	30	350	150

**Table 9:** Comparison of classification results.

Data sets	LSTM	EO-LSTM	TAFEO-LSTM
Banknote	91.89	92.14	**99.43**
Blood	71.56	79.67	**96.53**
Climate simulation	72.10	73.53	**80.99**
Indian	80.71	88.08	**96.49**
Pima	78.81	80.62	**80.71**
WDBC	89.47	93.39	**94.72**

**Table 10:** Parameter settings in the model.

Algorithms	Number of neurons	Batchsize	Maxepoch
LSTM	150	15	18
MPA-LSTM	156	12	13
WOA-LSTM	140	13	16
GWO-LSTM	150	14	18
EO-LSTM	162	13	15
TAFEO-LSTM	141	12	15

**Table 11:** Data of two wells.

Well name		Number of data	Number of reservoir data	Number of nonreservoir data
D1	Training well section	467	141	326
	Test well section	200	70	130

D2	Training well section	1867	270	1597
	Test well section	800	172	623

**Table 12:** Comparison of application performance for D1.

Algorithms	Accuracy (%)
LSTM	80.13
MPA-LSTM	84.77
WOA-LSTM	86.75
GWO-LSTM	90.63
EO-LSTM	92.72
TAFEO-LSTM	94.04

**Table 13:** Comparison of application performance for D2.

Algorithms	Accuracy (%)
LSTM	87.52
MPA-LSTM	88.89
WOA-LSTM	90.76
GWO-LSTM	87.52
EO-LSTM	91.39
TAFEO-LSTM	95.01

**Table 14:** Results of reservoir identification and comparison of models.

Model Name	Accuracy

Fisher different approach	76.00%
BP neural network	85.75%
ELM	91.13%
SVM	87.75%
CNN	93.25%
TAFEO-LSTM	95.01%

## Data Availability

The data used to support the findings of this study are available from the corresponding author upon request.
